# Reproduction Does Not Adversely Affect Liver Mitochondrial Respiratory Function but Results in Lipid Peroxidation and Increased Antioxidants in House Mice

**DOI:** 10.1371/journal.pone.0160883

**Published:** 2016-08-18

**Authors:** Annelise V. Mowry, Andreas N. Kavazis, Aubrey E. Sirman, Wayne K. Potts, Wendy R. Hood

**Affiliations:** 1 Department of Biological Sciences, Auburn University, Auburn, Alabama, United States of America; 2 School of Kinesiology, Auburn University, Auburn, Alabama, United States of America; 3 Department of Biology, University of Utah, Salt Lake City, Utah, United States of America; University of Alabama at Birmingham, UNITED STATES

## Abstract

Reproduction is thought to come at a cost to longevity. Based on the assumption that increased energy expenditure during reproduction is associated with increased free-radical production by mitochondria, oxidative damage has been suggested to drive this trade-off. We examined the impact of reproduction on liver mitochondrial function by utilizing post-reproductive and non-reproductive house mice (*Mus musculus*) living under semi-natural conditions. The age-matched post-reproductive and non-reproductive groups were compared after the reproductive females returned to a non-reproductive state, so that both groups were in the same physiological state at the time the liver was collected. Despite increased oxidative damage (p = 0.05) and elevated CuZnSOD (p = 0.002) and catalase (p = 0.04) protein levels, reproduction had no negative impacts on the respiratory function of liver mitochondria. Specifically, in a post-reproductive, maintenance state the mitochondrial coupling (i.e., respiratory control ratio) of mouse livers show no negative impacts of reproduction. In fact, there was a trend (p = 0.059) to suggest increased maximal oxygen consumption by liver mitochondria during the ADP stimulated state (i.e., state 3) in post-reproduction. These findings suggest that oxidative damage may not impair mitochondrial respiratory function and question the role of mitochondria in the trade-off between reproduction and longevity. In addition, the findings highlight the importance of quantifying the respiratory function of mitochondria in addition to measuring oxidative damage.

## Introduction

The notion that there is a tradeoff between reproductive investment and longevity is a central tenet of biology. The disposable soma theory of aging posits that this relationship arises because the allocation of energy to reproduction detracts from allocation to tissue maintenance [1]. Reproduction increases the demand for ATP produced by mitochondria, organelles that are also responsible for the production of reactive oxygen species (ROS) that damage intracellular lipids, proteins, and DNA [2]. This damage is thought to contribute to the decline in cell and tissue function that occurs with senescence (mitochondrial free radical theory of aging [2]). In this regard, it has been proposed that reproduction increases oxidative damage that may reduce the ability of cells to maintain function, which can negatively affect longevity [3–5]. But support for this assumption is equivocal. Recent reviews found no consistent relationship between reproduction and oxidative damage [6, 7].

Recently, research has shown that the assumption of a linear relationship between ROS production and ATP production was incorrect [7]. Indeed, the speed of electron movement through the electron transport chain (ETC) is tied to the redox state of the complexes. When the rate of oxygen consumption and ATP production slow, the rate of electron flow through the ETC also slows, and the ETC complexes become reduced. In this state, the probability that electrons escape the ETC and be converted to ROS is enhanced [8–10]. Moreover, studies also question whether oxidative damage is responsible for mitochondrial DNA mutations that lead to a decline in respiratory function [11, 12]. Few ecological or evolutionary studies have directly tested the effect of a trait assumed or known to induce oxidative damage, such as reproduction, on mitochondrial respiratory function. Without these data, it should not be assumed that oxidative damage is correlated with a negative impact on the ability of mitochondria to meet the respiratory demands of the cell or tissue being evaluated [13].

During pregnancy and lactation, the body undergoes dramatic physiological changes that are reversed after reproduction. Most investigators compare oxidative damage in reproductive versus non-reproductive animals [as reviewed by [6, 7]], but this approach makes it impossible to distinguish any irreversible costs of reproduction on the ability of tissues to support basic function (maintenance) from the reversible changes that enable reproduction. A more appropriate time to evaluate the impact of reproduction on maintenance is after reproduction, once the body returns to allocating resources to maintenance. While oxidative damage and antioxidants provide a measure of damage and maintenance, measuring mitochondrial respiratory function is critical to understanding the long-term functional impact of reproduction on longevity.

The aim of this study was to examine the impact of reproduction on liver mitochondrial respiratory function and oxidative stress, which is hypothesized to fuel the trade-off between reproduction and longevity. If oxidative damage is responsible for the reproduction-longevity trade-off, post-reproductive animals would display reduced mitochondrial respiratory function associated with accumulated oxidative stress.

## Materials and Methods

### Animals

We evaluated mitochondrial function in outbred lines of house mice, *Mus musculus*, founded by WKP [14]. Mice included in this study were 14 generations removed from the wild and housed in enclosures designed to mimic the conditions of mice living in a barn. Each enclosure was designed to match the natural home range (5 m^2^) and social group size (~ 10 adults) of wild mice [15]. The enclosures were divided between 2 wood buildings that each sit on a concrete slap; the buildings are covered and have hardware-cloth windows that excluded predators but exposed the mice to ambient temperatures.

### Experimental design

At sexual maturity (~2 months of age), each population was founded with 3 male and 5–7 female mice. Within the enclosures, mice were allowed to establish a natural social structure and breed at their own pace. Mice were offered *ad libitum* access to a 10% or 20% protein, isocaloric diet following the design of another project. We found no effect of diet on mitochondrial quality (t_17_ = 0.08, p = 0.94) and thus excluded diet from further consideration. Due to postpartum estrus, females commonly nurse while gestating their next litter. Pups were removed at 4 week of age to maintain population densities. Males were kept in the enclosures with the females until the females were 10–11 months old. When the females were 10–11 months, the males were removed to ensure that all females were non-reproductive at the time of tissue collection. All females were then euthanized for tissue collection at approximately 1 year of age. This duration allowed females that had recently mated to complete pregnancy (3 weeks), lactation (3 weeks), and allowed reproductive tissues to regress after weaning. Reproductive females produced up to 7 litters.

After founding the populations, the number of mice in each enclosure was reduced over time due to death or injury before the study ended. At the time males were removed, there were 2:5, 3:5, 2:5, 2:5, 1:4,1:6 in the reproductive enclosures and 1:3 and 1:3 (M:F) in the non-reproductive (defined below) enclosures. In two enclosures, pups were never observed during daily checks; we refer to these females as non-reproductive. It is possible that some females experienced gestation and resorbed their fetuses or produced litters that were lost within the first 24 hours (between daily checks) due to cannibalism, but none were observed lactating. Because both of these enclosures ultimately had one male and several females, we speculate that the single dominant male in both enclosures was sterile. The body condition of these females was good and the condition of the non-reproductive females did not appear to differ from females in the other enclosures.

At 1 year of age, females were anesthetized with an overdose of isoflurane and then decapitated. Because mice were collected simultaneously from 1 or 2 enclosures and we could not measure respiratory function of mitochondria from more than 4 mice simultaneously, mitochondria were isolated from the livers of 2 to 4 randomly selected female mice per enclosure, resulting in a sample size of 15 post-reproductive and 4 non-reproductive females.

All animal procedures and housing conditions described herein were approved by the Auburn University IACUC (PRN# 2102–2104).

### Isolated mitochondria isolation and measurements

Immediately following euthanasia, the liver was removed from each mouse and mitochondria were isolated as detailed in Sewell et al [16]. Briefly, livers were weighed and put into 10 volumes of a solution made up of 250 mM sucrose, 5 mM HEPES, and 1 mM EGTA and minced with scissors. This minced tissue was further homogenized with a Potter-Elvhjem PTFE pestle and glass tube. The resulting homogenate was centrifuged for 10 min at 500 g at 4°C, pelleting the cellular debris. The supernatant was then decanted through cheesecloth and then centrifuged for 10 min at 3,500 g at 4°C, pelleting the mitochondrial fraction. The supernatant was removed and the pellet resuspended in the sucrose solution. This solution was centrifuged for 10 min at 3,500 g at 4°C, the supernatant discarded and the final mitochondrial pellet suspended in 250 μl of a solution made up of 220 mM mannitol, 70 mM sucrose, 10 mM Tris+HCl, and 1 mM EGTA, at a pH of 7.4 [17]. Isolated mitochondria (20 μL) were incubated in 1 ml of respiration buffer at 37°C and respiration rates were determined polarographically (Oxytherm, Hansatech Instruments, UK) following Messer et al [18] using 2 mM pyruvate and 2 mM malate. The maximal respiration (state 3), defined as the rate of respiration in the presence of ADP was initiated by adding 0.25 mM ADP to the respiration chamber containing mitochondria and respiratory substrates. State 4 respiration was recorded following the phosphorylation of ADP. State 3 and state 4 respiration rates were normalized to mitochondrial protein concentration and expressed as O_2_/mg mitochondrial protein/min. The respiratory control ratio (RCR) was calculated by dividing state 3 by state 4 [13].

### Western blotting

The protein levels of the antioxidants copper-zinc superoxide dismutase (CuZnSOD; GTX100554; GeneTex, Irvine, CA), manganese superoxide dismutase (MnSOD; GTX116093; GeneTex), glutathione peroxidase 1 (GPX-1; GTX116040; GeneTex), catalase (GTX110704; GeneTex), and the marker of lipid peroxidation (4-Hydroxynonenal; 4-HNE; ab46545; Abcam, Cambridge, MA) were measured by Western blotting [19] in isolated liver mitochondria [20, 21]. The protein content of these blots was normalized to α-tubulin (GTX112141; GeneTex) levels (the loading and transfer control) since alpha-tubulin is an inherent component of mitochondrial membranes [22]. This method does not address the purity of the mitochondrial isolation, as α-tubulin is not unique to mitochondria; however, centrifugation at 3,500 g during mitochondrial isolation has been shown to minimize contamination by peroxisomes and other organelles [23]. A chemiluminescent system was used to visualize marked proteins (GE Healthcare Life Sciences, Pittsburgh, PA). Images were taken with the ChemiDocIt Imaging System (UVP, LLC, Upland, CA).

### Statistical analysis

Statistical analyses were completed using R version 3.0.3 [24]. Post-reproductive and non-reproductive groups were compared using two-sample t-test. Significance was established at p ≤0.05.

## Results

### Mitochondrial oxidative phosphorylation

**Fig 1:**
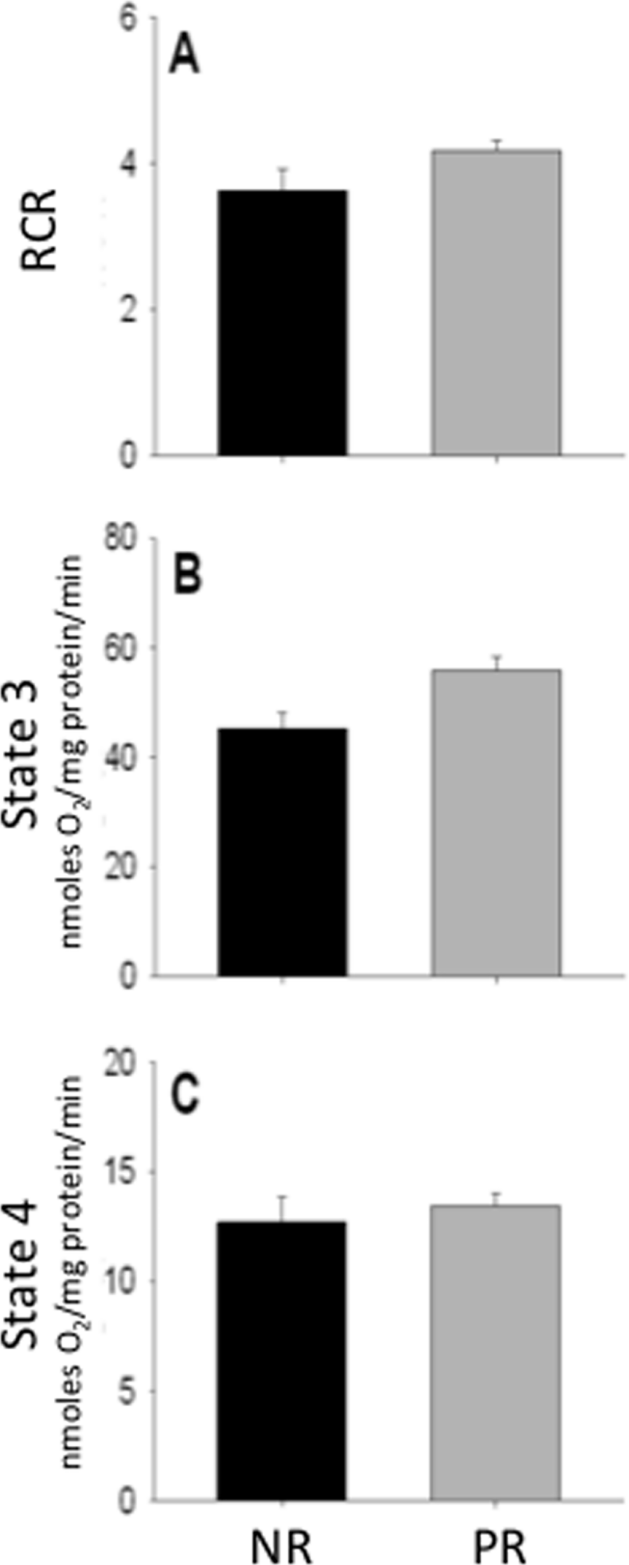
Liver mitochondria respiratory function for non-reproductive (PL) and post-reproductive (PR) female house mice. RCR (A), state 3 (B), and state 4 respiration (C) in liver mitochondria of non-reproductive and post-reproductive female mice are given. State 3 and state 4 respiration rates were measured polarographically and normalized to mitochondria content. RCR was calculated as the ratio of state3/state 4 respiration. Standard error bars are given and representative blots depicted for each graph. Non-reproductive, n = 4; Post-reproductive, n = 15. No significant differences were observed between groups.

To determine how reproduction affects mitochondrial efficiency, the respiratory control ratio of isolated mitochondria was measured. Specifically, we measured the respiratory function of the mitochondria by evaluating their respiratory control ratio (RCR), a measure of state 3 (maximum metabolic rate) to state 4 (basal metabolic rate) respiration. While alternative measures have been described [25], RCR is thought to be a particularly valuable measure of mitochondrial function because it is responsive essentially to all changes in the functionality of the ETC [13]. RCR of liver mitochondria was similar in post-reproductive compared to non-reproductive females, although there was a trend for higher RCR in post-reproductive mice (t_17_ = 1.80; p = 0.089; Fig 1A). When state 3 and state 4 respiration were considered independently, the difference in RCR between groups appears to be driven by state 3 respiration, or maximal respiration capacity, as the livers of females that reproduced display state 3 respiration that bordered on higher than non-reproductive controls (t_17_ = 2.02; p = 0.059; Fig 1B). Furthermore, state 4 respiration, i.e. basal respiratory rate, was similar between the two groups (t_17_ = 0.602; p = 0.555; Fig 1C).

### Oxidative damage

**Fig 2:**
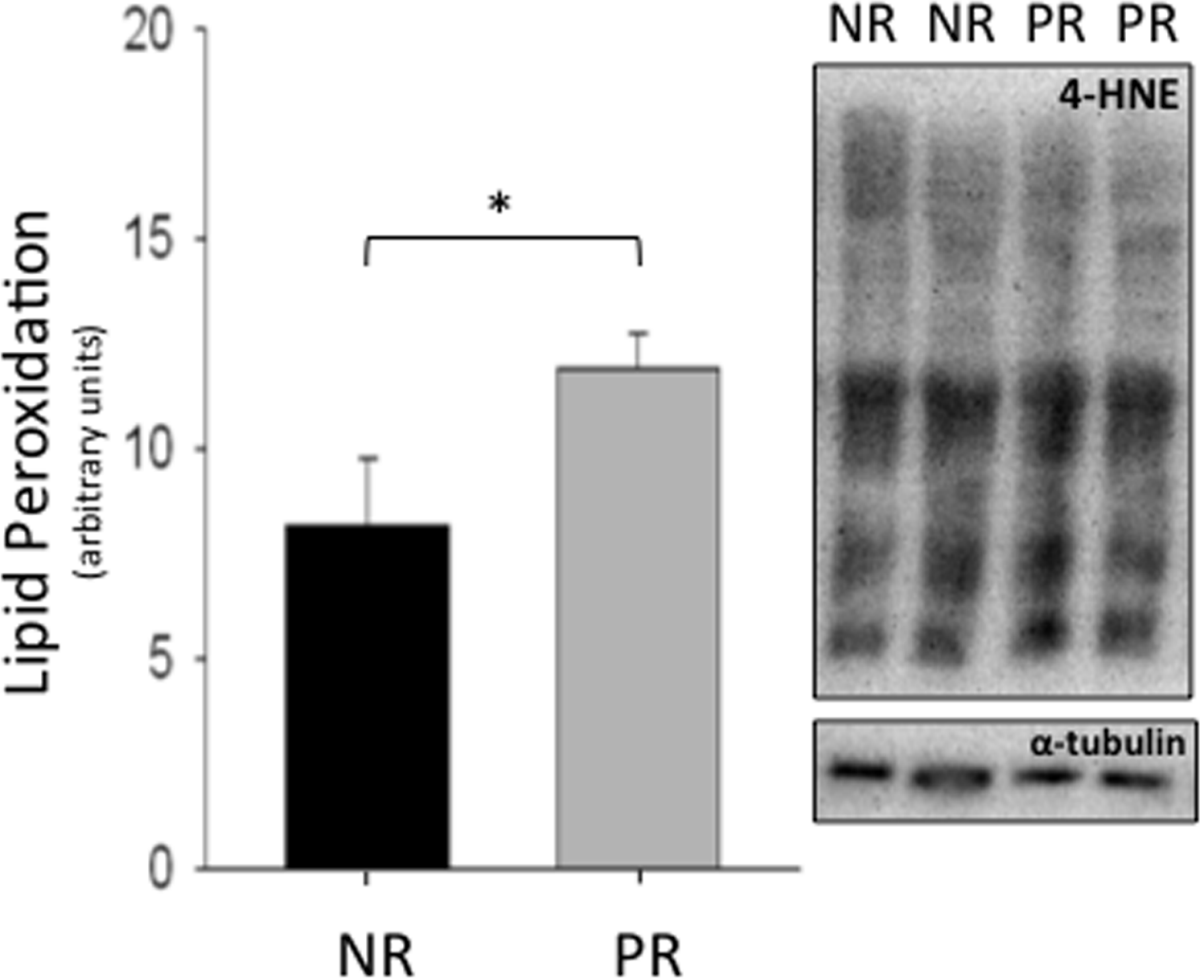
Liver peroxidation levels for non-reproductive (NR) and post-reproductive (PR) female house mice. 4-hydroxynonenal (4-HNE) levels (whole lane) from isolated liver mitochondria of non-reproductive and post-reproductive female mice are given. Each graph included standard error bars and representative blots. Concentrations were quantified by protein densities of Western blot bands and normalized by concentrations of α-tubulin. Non-reproductive, n = 4; Post-reproductive, n = 15; * p = 0.050, significantly different compared to non-reproductive group.

A measure of lipid peroxidation, 4-HNE, was used to assess damage from oxidative stress [26]. The liver mitochondria of post-reproductive females exhibited significantly more lipid peroxidation than non-reproductive controls (t_17_ = 2.07; p = 0.050; Fig 2).

### Antioxidants

**Fig 3:**
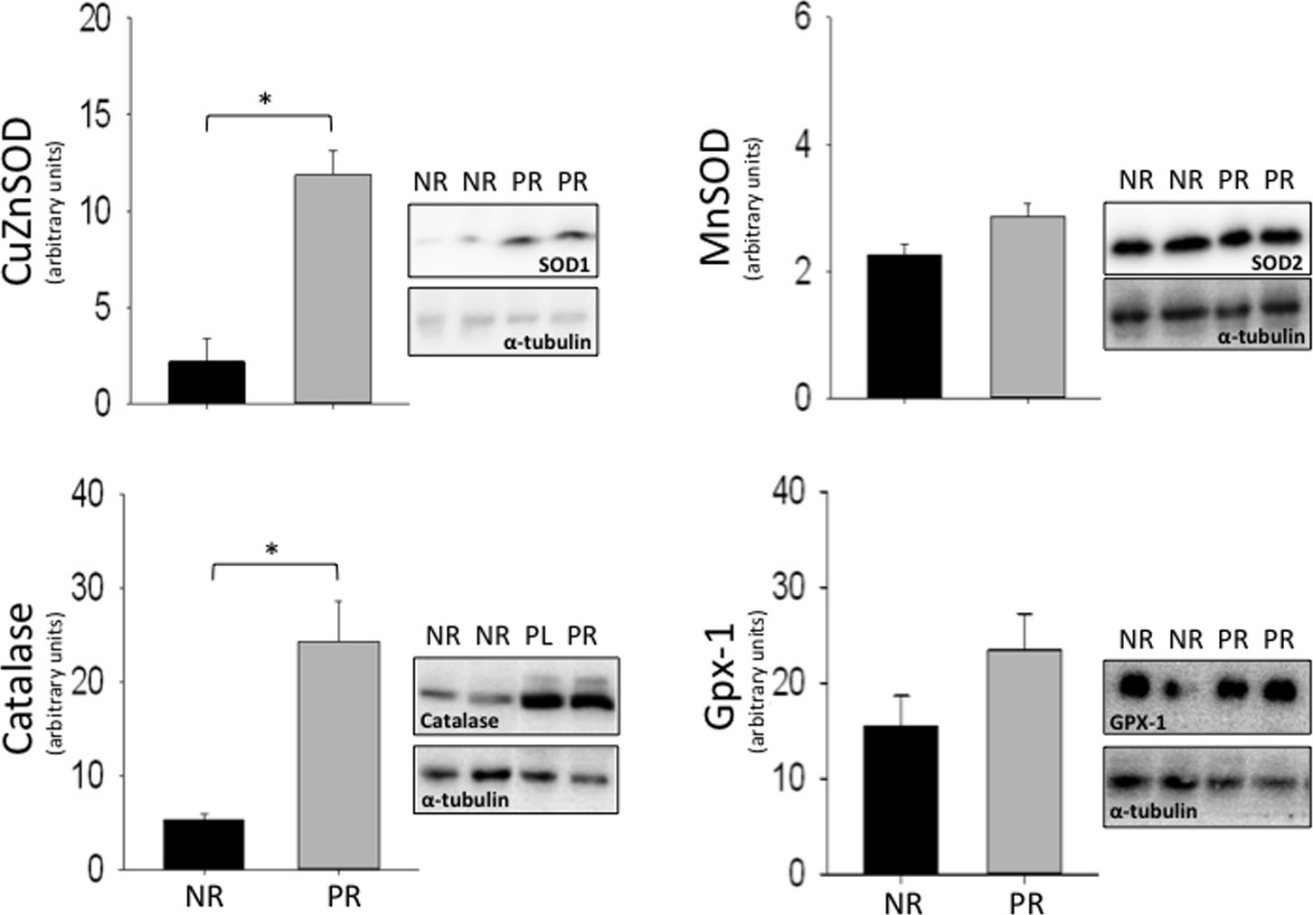
Relative antioxidant levels for non-reproductive (NR) and post-reproductive (PR) female house mice. Levels of Copper-zinc superoxide dismutase (CuZnSOD), manganese superoxide dismutase (MnSOD), catalase, and glutathione peroxidase 1 (Gpx-1) in isolated liver mitochondria of non-reproductive and post-reproductive female mice are given. Each graph included standard error bars and representative blots. Concentrations were quantified by protein densities of Western blot bands and normalized by concentrations of α-tubulin. Non-reproductive, n = 4; Post-reproductive, n = 15; * p<0.05, significantly different compared to non-reproductive group.

The effects of reproduction on the relative level of several intra-mitochondrial antioxidants were quantified. Specifically, CuZnSOD and MnSOD dismutate superoxide to hydrogen peroxide and oxygen [20, 21]. In turn, intra-mitochondrial catalase and glutathione peroxidase 1 (GPX-1) convert hydrogen peroxide into water and oxygen [21]. Females that reproduced had significantly higher CuZnSOD (t_17_ = 3.73; p = 0.002; Fig 3) and catalase (t_17_ = 2.22; p = 0.040; Fig 3) than non-reproductive female, while MnSOD (t_17_ = 1.43; p = 0.169; Fig 3) and GPX-1 (t_17_ = 1.02; p = 0.320; Fig 3) were similar between groups.

## Discussion

The mitochondrial free radical theory of aging suggests that steady deterioration associated with aging is caused by the accumulation of ROS damage. Molecules that are damaged include mtDNA [27]. While most damaged mitochondria are destroyed, remaining mitochondria replicate [28, 29]. Turnover occurs every 3–4 days, contributing to an increasingly mutated mitochondrial population over a lifetime [29, 30]. Therefore, the energetic demands of reproduction are predicted to accelerate the decline in mitochondrial respiratory function that is associated with senescence.

Contrary to this prediction, we found that reproduction did not have a lasting effect on the respiratory capacity of liver mitochondria. Indeed, we found a trend, which warrants follow-up studies due to the sample size used, suggesting higher RCR in post-reproductive than non-reproductive females. This trend counters a previous report that livers from mice at peak-lactation do not have different RCR and state 3 respiration is lower (Clc+CII normalized to citrate synthase) than in virgin females [31]. Unlike the current study, Pichaud et al’s [31] wild-derived mice were housed in standard rodent boxes and were not re-mated immediately after birth. Thus, Pichaud et al’s mice did not experience concurrent gestation and lactation, as often occurs in mice in natural populations [31]. Furthermore, as Pichaud et al collected data at peak lactation and thus, their findings reflect the physiological changes that allow the liver to meet the energetic and fatty acid demands of milk production [31]. Changes that support reproductive demand cannot be uncoupled from the impact that reproduction has on future organ capacity during the reproductive event. Thus, organ specific mitochondrial function may display relatively high plasticity as females transition between reproductive and maintenance state. Our results show that post-reproductive females maintain the basal respiration rate of their mitochondria and show similar or perhaps even increased maximum mitochondrial respiratory capacity (Fig 1B), suggesting that there may be no negative impact of reproduction on mitochondrial function. It is important to recognize that these mitochondrial measurements were completed *in vitro*, and thus the relationship between mitochondrial respiratory function and reproduction may warrant further investigation *in vivo*.

Our results indicate that the membrane lipids of liver mitochondria in post- reproductive females sustained more oxidative damage than non-reproductive individuals (Fig 2). Due to rapid mitochondrial turnover, the liver mitochondria evaluated are the decedents of those mitochondria present during reproduction. While it is possible that the oxidative state of the animals was product of recent conditions, ROS induced damage to mtDNA over the life of an animal can alter mitochondrial proteins, reduce coupling, and as a result increase rate of ROS production [32]. While the mice in this study must be considered relatively old since they were maintained under naturalistic conditions, a significant accumulation of mtDNA mutations may be necessary before mitochondrial respiratory function is detected [33, 34]. It is also possible that the levels of ROS produced in both post-reproductive and non-reproductive mice were sufficient to upregulate antioxidants and repair mechanisms [35–37], but insufficient to increase rate of senescence, as is predicted by the theory of mitochondrial hormesis [35, 38].

Antioxidants neutralize free radicals to prevent damage to macromolecules [39]. Specifically, CuZnSOD and MnSOD convert superoxide to hydrogen peroxide and oxygen, and catalase and GPX-1 catalyze the conversion of hydrogen peroxide to water and oxygen [40–42]. We found that post-reproductive females displayed higher liver mitochondria protein levels of CuZnSOD and catalase and higher oxidative damage than non-reproductive controls. Assuming that an increase in protein abundance of these two antioxidant enzymes (i.e., CuZnSOD and catalase) translates into increased enzyme activity and detoxification of oxidants, it is predicted that liver mitochondria from post-reproductive females would eliminate more oxidants than non-reproductive females. This suggests that oxidant production (although not measured in this study) is higher in post-reproductive females, as high intra-mitochondrial antioxidants were insufficient to keep oxidative damage at the lower level observed in non-reproductive females. However, since the purity of the mitochondrial fraction was not measured, it is possible that antioxidants from other subcellular compartments could be contributing to these changes. Previous studies have reported higher antioxidant production, but lower oxidative damage at peak-lactation in female house mice compared to non-reproductive females [6, 43]. Our study housed mice in enclosures designed to mimic naturally occurring social groups. It is possible that the oxidative damage in post-reproductive females in this study is higher compared to reported oxidative damage in reproductive mice because our study houses mice in semi-natural enclosures that elicit stress from social interactions [43].

Finally, we note that associated with this being an opportunistic study, tissues could only be collected from one organ and thus, provides a limited snapshot of how the body responds to reproduction. The digestive track also displays dramatic hyperplasia in response to reproduction in rodents and thus, it is possible the reproduction could also have a significant effect on the function of this, and potentially other organs [44, 45]. Although the mammary gland typically displays dramatic changes in response to reproduction, its mass is minimal in non-reproductive female rodents and thus, the respiratory function of mammary mitochondria can’t be compared between reproductive and non-reproductive rodents using the methods described herein.

## Conclusion

With our unique experimental design, comparing non-reproductive and experienced breeders in a post-reproductive state, we determined that reproduction does not adversely impact liver mitochondrial quality at the age of 1 year. Although this finding should be consider preliminary, these results question the nature of the trade-off between reproduction and longevity and highlight the importance of measuring mitochondrial respiratory function when measuring oxidative damage. The results of this study are consistent with previous findings suggesting that oxidative damage may not be associated with increased rate of senescence [11, 12, 46].
